# Supplementary material for: Intercepted Mosquitoes at New Zealand’s Ports of Entry, 2001 to 2018: Current Status and Future Concerns
Source: Trop Med Infect Dis. 2019 Jul 5;4(3):101. doi: 10.3390/tropicalmed4030101 (PMC6789606; doi:10.3390/tropicalmed4030101)
Supplement: Supplementary file 1 [file tropicalmed-04-00101-s001.pdf]

**Supplementary S1:** New Zealand total gross weight of sea imports, total number of used tyre imports from all sources and from Japan, and total used machinery imports from all sources, 2001—2017

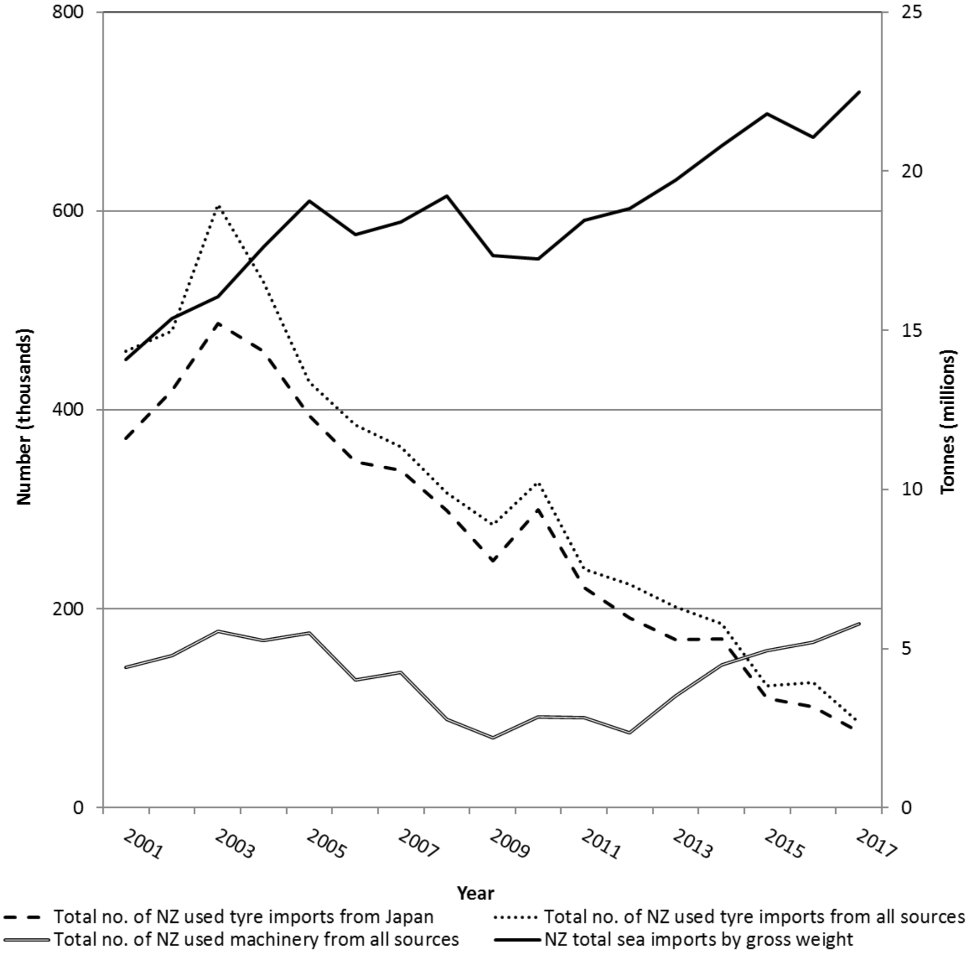


**Supplementary S2: (A)** (Left axis): Total number of passenger flights to New Zealand from all overseas ports and from ports where Ae. aegypti is established. (Right axis): New Zealand total gross weight of airfreight imports. **(B)** Total number of passenger flights to New Zealand from major overseas ports where Ae. aegypti is established

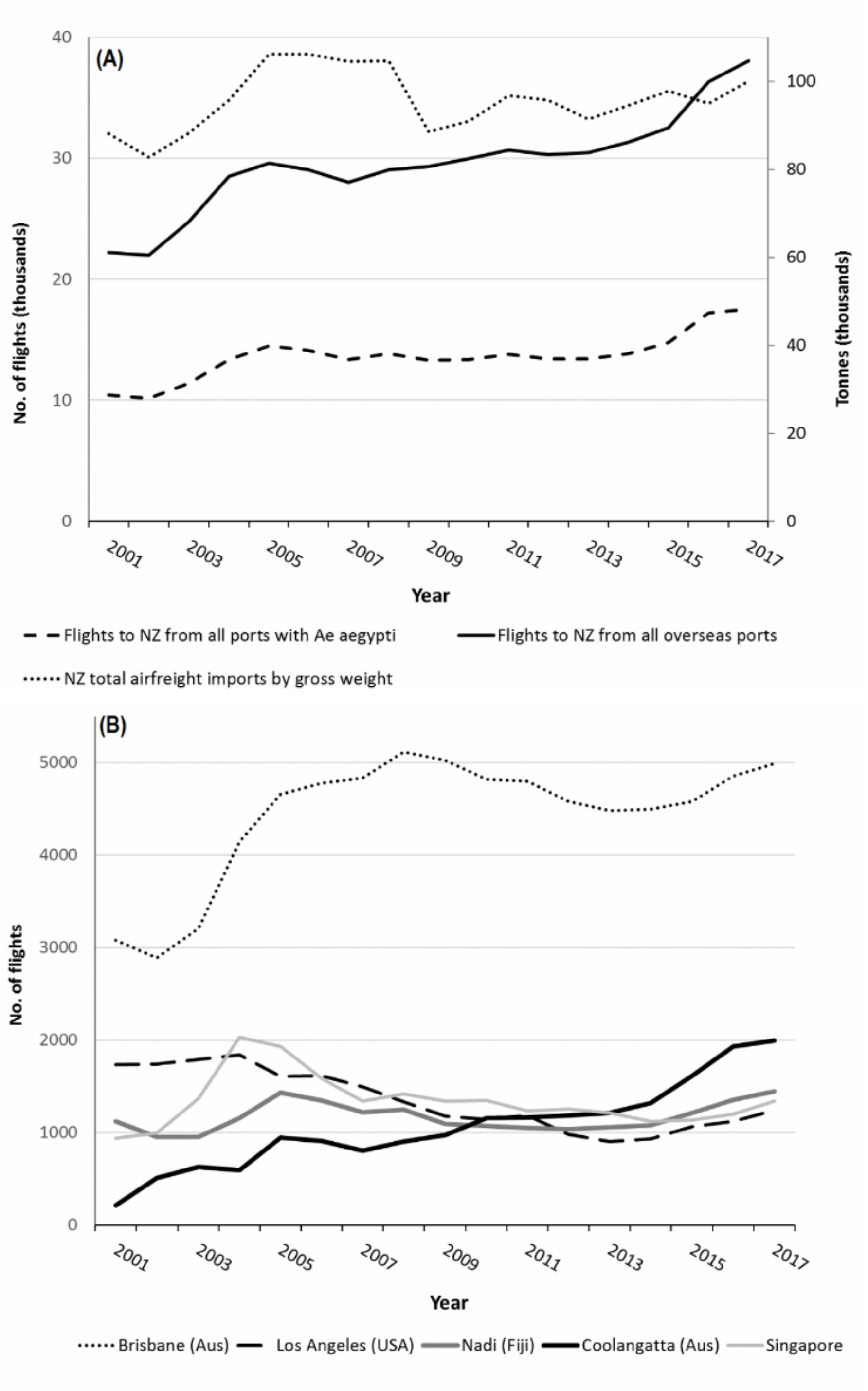


**Supplementary S3:** Imports of used tyres by New Zealand, 2001—2017 showing countries where *Ae. aegypti* and *Ae. albopictus* are present

| Country of origin | Used tyres | | Used vehicles | | Presence or absence | |
|---|---|---|---|---|---|---|
| | Quantity | % | Quantity | % | *Ae. aegypti* | *Ae.albopictus* |
| **Japan** | 4,703,435 | 87.7 | 1,959,112 | 86.61 | - | + |
| **Germany** | 264,294 | 4.9 | 184,852 | 8.17 | - | + |
| **Australia** | 147,414 | 2.7 | 16,832 | 0.74 | + | - |
| **Taiwan** | 41,680 | 0.8 | 24 | 0.00 | + | + |
| **S. Korea** | 39,811 | 0.7 | 5,725 | 0.25 | - | + |

| | | | | | | |
|---|---|---|---|---|---|---|
| **Singapore** | 34,107 | 0.6 | 679 | 0.03 | + | + |
| **Netherlands** | 24,055 | 0.4 | 162 | 0.01 | - | - |
| **China** | 22,538 | 0.4 | 276 | 0.01 | + | + |
| **USA** | 17,388 | 0.3 | 36,555 | 1.62 | + | + |
| **UK** | 13,958 | 0.3 | 30,269 | 1.34 | - | - |
| **UAE** | 12,029 | 0.2 | 29 | 0.00 | - | - |
| **Italy** | 11,529 | 0.2 | 3,353 | 0.15 | - | + |
| **Thailand** | 9,394 | 0.2 | 686 | 0.03 | + | + |
| **Malaysia** | 5,403 | 0.1 | 103 | 0.00 | - | + |
| **Hong Kong** | 4,401 | 0.1 | 146 | 0.01 | - | + |
| **Belgium** | 3,283 | 0.1 | 671 | 0.03 | - | + |
| **France** | 3,207 | 0.1 | 8,903 | 0.39 | - | + |
| **Spain** | 1,588 | 0 | 519 | 0.02 | - | + |
| **Switzerland** | 1,106 | 0 | 35 | 0.00 | - | + |
| **Brazil** | 714 | 0 | 9 | 0.00 | + | + |
| **Jordan** | 559 | 0 | 105 | 0.00 | - | + |
| **Finland** | 89 | 0 | 7 | 0.00 | - | - |
| **Argentina** | 75 | 0 | 8 | 0.00 | + | - |
| **Indonesia** | 46 | 0 | 11 | 0.00 | + | + |
| **Fiji** | 34 | 0 | 36 | 0.00 | + | + |
| **Sweden** | 32 | 0 | 11,075 | 0.49 | - | - |
| **South Africa** | 16 | 0 | 661 | 0.03 | + | - |
| **Canada** | 7 | 0 | 220 | 0.01 | - | - |
| **Papua New Guinea** | 6 | 0 | 9 | 0.00 | + | + |
| **Botswana** | 5 | 0 | 0 | 0.00 | - | - |
| **Luxembourg** | 4 | 0 | 1 | 0.00 | - | - |
| **Slovakia** | 4 | 0 | 46 | 0.00 | - | + |
| **India** | 2 | 0 | 104 | 0.00 | - | + |
| **Philippines** | 1 | 0 | 5 | 0.00 | - | + |
| **Zimbabwe** | 1 | 0 | 0 | 0.00 | - | - |
| **Other** | 0 | 0 | 731 | 0.03 | | |
| Total | **5,362,215** | **100** | **2,261,959** | **100** | **17** | **22** |

Present +

Absent -

**Supplementary S4:** National concerns and development of responses for mosquito interceptions

In early 1993, the need for updated biosecurity measures towards mosquito interceptions in New Zealand was highlighted when *Ae albopictus* larvae were found in a wet used tyre shipment from Japan. This finding was consistent with the steady increase in used tyre importations between 1980 and 1990. As a quick response, from February 1993, all used tyre imports became subject to inspections. Later in the same year, compulsory offshore methyl bromide fumigation was required [1, 2]. In mid-1993, the Biosecurity Act was introduced to update laws and regulations relating to protection of New Zealand from imported organisms. The "Act" covers pre-border risk management and standard setting, border management readiness and response and long-term pest management. To test the updated measures, another extensive survey was undertaken between spring 1993 and autumn 1994, focusing on artificial breeding habitats, mainly used tyres and machinery. The survey showed negative results for any exotic *Aedes* mosquitoes [3].

In 1996, the Ministry of Health commissioned a review of the New Zealand programme for exclusion of exotic mosquitoes of public health significance [4]. Kay [4] reported many flaws in the programme, including an overall underdevelopment with a lack of systematic independent examination, a general deficiency of entomological expertise dealing with mosquitoes, and a non-compliance of the New Zealand authorities with the International Health Regulations at international points of entry. One year after the Kay review, the establishment of *Ae camptorhynchus* in New Zealand was discovered and then subjected to an 11-year successful eradication programme began initiated in January 1999 and costing $71 million [5].

Kay's recommendations were to set new standards and upgrade port surveillance activities. Between 1998 and 2000, the Ministry of Health worked with MPI (the former Ministry of Agriculture and Forestry, MAF) to introduce new guidelines and strategies, and improvement policies for exotic mosquito surveillance. This included the incorporation of import health standards and controls in relation to used tyres, and used machinery, disinsection of aircraft, responses to suspected interceptions of exotic mosquitoes at international airports, sea ports and transitional facilities [6]. A year later, New Zealand BioSecure Entomology Laboratory was established to provide entomological (mainly mosquito) identification services and technical advice for the Ministry of Health. Biosecurity New Zealand developed Import Health Standards for risk goods, including new and used vehicles, machinery and tyres. Import Health Standards state the requirements that must be met by importers to obtain biosecurity clearance. The requirements are regularly updated considering the scientific research advances and the infestation situation of the exotic vector species in the exporting countries. [2]. In 2007, the Biosecurity Science Strategy discussed the future priorities for biosecurity-related science and highlighted the central role of research in biosecurity.

**References**


1. Laird, M., et al., *Japanese Aedes albopictus among four mosquito species reaching New Zealand in used tires.* Journal of the American Mosquito Control Association-Mosquito News, 1994. **10**(1): p. 14-23.
2. Biosecurity New Zealand. Ministry of Agriculture and Forestry Wellington New Zealand, *Import risk analysis: vehicle and machinery*. 2007. p. 364.
3. Laird, M., *Background and findings of the 1993-94 New Zealand mosquito survey.* New Zealand Entomologist, 1995. **18**(1): p. 77-90.
4. Kay, B., *Review of New Zealand's programme for exclusion and surveillance of exotic mosquitoes of public health significance.* Report to the Ministry of Health, Wellington, 1997.
5. Kay, B. and R. Russell, *Mosquito Eradication: The Story of Killing Campto*. 2013: CSIRO Publishing.
6. Mariana Musicante (personal communications), *Principal Entomologist, NZBioSecure Entomology Laboratory (NZBEL)*, S. Ammar, Editor. 2018.
